# Cortical reorganization after cochlear implantation for adults with single-sided deafness

**DOI:** 10.1371/journal.pone.0204402

**Published:** 2018-09-24

**Authors:** Elsa Legris, John Galvin, Sylvie Roux, Marie Gomot, Jean-Marie Aoustin, Mathieu Marx, Shuman He, David Bakhos

**Affiliations:** 1 Université François-Rabelais de Tours, CHRU de Tours, UMR-S1253, Tours, France; 2 Ear Nose and Throat department, Tours, France; 3 House Ear Institute, Los Angeles, CA, United States of America; 4 Ear Nose and Throat department, Toulouse, France; 5 Department of Otolaryngology–Head and Neck Surgery, The Ohio State University, Columbus, OH, United States of America; Universidad de Salamanca, SPAIN

## Abstract

**Background:**

Adults with single sided deafness (SSD) have lost binaural function, which limits sound source localization, speech understanding in noise, and quality of life. For SSD patients, restoration of bilateral auditory input is possible only with a cochlear implant (CI). In this study, cortical auditory evoked potentials (CAEPs) and behavioral performance were measured in left-implanted (SSD-CI-L) and right-implanted (SSD-CI-R) patients before and after cochlear implantation. We hypothesized that improvements in behavioral performance would be accompanied by changes in CAEPs after cochlear implantation.

**Design:**

Prospective longitudinal study.

**Setting:**

Tertiary referral center.

**Method:**

Nine right-handed adult SSD CI patients participated in the study. CAEPs were recorded before cochlear implantation and at 6 and 12 months post-implantation. CAEPs were elicited using speech stimuli (/ba/) delivered in sound field at 70 dBA. Global field power (GFP) latency and amplitude were calculated for P1, N1 and P2 peaks at each test session. CAEP were analyzed at frontocentral (Cz) and temporal (P7, P8, T7 and T8) and mastoid electrodes (M1 and M2) contralateral to the CI ear. Behavioral measures (sentence recognition in noise, with and without spatial cues) were collected at the same test sessions as for CAEPs. Speech performance and CAEPs were also measured in a control group of normal-hearing (NH) subjects.

**Results:**

While increased N1 amplitude was observed in the scalp potential maps for GFP and Cz for SSD-CI-L patients after implantation, the changes were not statistically significant. Peak CAEP amplitude at electrodes to contralateral to the CI ear increased after cochlear implantation for all SSD-CI patients, but significant increases were observed only for mastoid sites. Peak latencies for some components at temporal and mastoid sites remained significantly longer than for the NH control group, even after cochlear implantation. For SSD-CI-R patients, P2 peak amplitude for baseline GFP and Cz was significantly lower than for the NH control group. A significant improvement for speech understanding in noise was observed at 12 months post-implantation when speech was presented to the CI ear and noise to the non-implanted ear.

**Conclusion:**

After cochlear implantation, speech understanding significantly improved when speech and noise were spatially separated. The increased N1 amplitude for SSD-CI-L patients and the increased bilateral activation for all SSD-CI patients may reflect cortical reorganization and restoration of binaural function after one year of experience with the CI. However, because of the limited number of SSD patients, significant changes in cortical activity after cochlear implantation were often difficult to observe.

## Introduction

Binaural perception is important everyday listening environments, allowing for sound source localization and speech understanding in noisy environments, especially when speech and noise are spatially separated. Binaural function depends on the activity of binaural neurons in the central auditory pathways, especially at the levels of the superior olivary complex, the nuclei of the lateral lemniscus, and the inferior colliculus [1]. Impairment of binaural function, as in cases of single-sided deafness (SSD) or asymmetrical hearing loss (AHL) can negatively impact localization, speech understanding in noise, and quality of life (QoL) [2,3]. The prevalence of SSD is estimated to affect 3–6% of the general population [4]. The most common etiologies in adults are sudden sensorineural hearing loss (SNHL), Meniere’s disease, temporal bone traumatism, ototoxicity (drugs and/or noise) and vestibular schwannoma [5].

Morphologic and functional effects of profound acquired unilateral deafness on the central auditory pathways have been investigated by several studies. Using functional magnetic resonance imaging (fMRI), some studies found that adults with unilateral deafness on the right side have decreased gray matter volume bilaterally in the posterior cingular gyrus and in the gyrus parahippocampic, as well as in the right lingual gyrus [6]. Zhang et al. (2015) reported more changes in the default mode network on the left side than on the right side for people with deafness [7]. Previous studies have also reported central auditory system reorganization due to unilateral deafness. Compared to normal hearing (NH) adults, patients with late onset unilateral deafness exhibited altered hemispheric asymmetries when monaural auditory stimulation delivered the unimpaired ear in terms of cortical auditory evoked potentials (CAEPs) [8], magnetoencephalography (MEG) [9], and fMRI [10,11].

For SSD patients, common interventions include contralateral routing of sound (CROS) or bone-conduction devices (BCDs) [12–14]. However, these approaches simply deliver sound to the unimpaired ear and do not restore binaural function. For SSD and AHL patients, restoration of binaural inputs is possible with a cochlear implant (CI). However, SSD and AHL often do not meet candidacy requirements for cochlear implantation (typically, severe-to-profound bilateral deafness). Unlike the CROS or BCDs, the CI directly stimulates surviving auditory neurons in the impaired ear. Arndt et al. (2017) [15] found better localization and speech understanding in noise for adult SSD subjects fitted with a CI than a CROS or BCD. Many studies with adult SSD patients have shown improved localization, speech understanding in noise, QoL, as well as reduced tinnitus severity after cochlear implantation [16–19].

A well-established approach for studying the time course of cortical responses to auditory stimulation is to record CAEPs, which are the summed electrical potentials of large fields of coherently activated neurons as recorded from the scalp, and represent auditory processing in the cerebral cortex. Penfield and Jasper [20] first localized the auditory cortex in the superior temporal gyrus by means of direct cortical stimulation. CAEPs are composed of multiple components. Adults show four waves with a positivity (P1) at about 50 ms, a negativity (N1) at about 100 ms, another positivity (P2) at about 200 ms [21–23] and another negativity (N250) at about 25Bases. P1 is thought to reflect early pre-perceptual processing of acoustic features [24]. Adult N1 would be sensitive to onset features, such as the slope and amplitude of the rise and fall of the auditory stimulus, and correlates with detection [25]. In contrast, P2 reflects the finer grained properties of the stimulus rather than its onset properties (e.g., speech versus non-speech, or familiar versus unfamiliar talkers, etc.) [26]. N250 allows for further assessment of cortical activity involved in processing of speech stimuli [27]. The most-studied component in adults is the N1. Neurophysiological studies conducted after removal of temporal lobe lesions have detected N1 generators in the auditory superior temporal cortex [25]. Its topography is characterized by a negative potential field over the frontocentral scalp areas and positive potentials at mastoid sites; this polarity reversal is typical of activity in the auditory cortex [28]. In posterior temporal areas, the activity is described by the T-Complex and has 3 major components: two negative peaks Na and Tb, and positive peak Ta [29–33]. The T-complex has been described as a useful clinical measure of higher-level auditory function in children [34] and adults [35]. CAEPs have been used with considerable success to assess the integrity of the auditory system in pediatric and adult CI users [36–41].

The objective of this longitudinal study was to investigate potential changes in auditory cortical responses and in speech perception following cochlear implantation in adult SSD patients. Similar to previous SSD studies, we predicted that speech understanding in noise would improve after implantation, especially when speech was presented to the CI ear and noise to the acoustic hearing ear. Given this expected improvement in behavioral performance, we were interested in how cortical activity might change after cochlear implantation. Data from pediatric SSD studies suggest changes in cortical responses after implantation [42–44]. For adult SSD patients, it is unclear how responses might change or the time course for changes to occur. In this study, CAEP and behavioral data were measured before cochlear implantation and at 6 and 12 months post-implantation. Data were also collected in a normal hearing (NH) group to compare to SSD patient data. Our main research question was: does restoration of binaural function with cochlear implantation lead to cortical reorganization in adult SSD patients? We expected changes in cortical responses as SSD patients gained experience with their CI, mostly on the side contralateral to the implanted ear.

## Methods

### Participants

Nine adults (5 women and 4 men), right-handed, French native speakers with acquired SSD (SSD-patients) were included in this longitudinal study. All patients had at least one year of experience with their CI. Subjects were recruited from the CI unit of the otolaryngology department at University Hospital of Tours, France. None of the participants had retro-cochlear pathology according to cranial MRI and all had a mini mental state score of 30/30. Table 1 shows patient demographic information. All subjects had profound SNHL in one ear; the mean unaided air pure tone average (PTA) threshold across 0.5, 1.0, 2.0 and 3.0 kHz was >70 dB HL in the ear to be implanted. Aided disyllable French word recognition [45] was <50% at 60 dB SPL in the ear to be implanted. PTA thresholds were ≤20 dB HL in the non-implanted ear. Across all subjects, the mean age at implantation was 60±7 years and the mean duration of deafness was 13±21.4 years. Five subjects were implanted on the left side (SSD-CI-L) and four were implanted on the right side (SSD-CI-R). A Mann Whitney test showed no significant difference (p = 0.900, U = 10) for age at implantation between SSD-CI-L (mean = 59±8 years) and SSD-CI-R patients (mean = 61±5 years). A Mann Whitney test also showed no significant difference (p = 0.400, U = 10) for duration of deafness between SSD-CI-L (mean = 6±8 years) and SSD-CI-R patients (mean = 11±13 years). In terms of etiology of deafness, six subjects had sudden hearing loss, one had Meniere’s disease, and for two subjects, the etiology was unknown. Approximately two weeks after surgery, the CI processor was activated and fit according to standard clinical procedures for bilaterally deaf CI patients. All SSD CI patients received intensive auditory training during the first year of CI use. None of the participants wore a hearing aid in their non-implanted ear. SSD patient demographic information is shown in Table 1.

**Table 1:** Demographic information for the present SSD-CI subjects.

Subject	Gender	Age (yrs)	Dur deaf (yrs)	Etiology	Non-CI ear PTA (dB HL)	CI ear PTA (dB HL)	CI ear	CI device
**S1**	M	53	1.5	Unknown	19	120	L	CI522 ^C^
**S2**	F	66	5.5	Sudden hearing loss	5	83	L	CI512 ^C^
**S3**	F	65	2	Sudden hearing loss	20	73	L	CI512 ^C^
**S4**	M	48	2	Sudden hearing loss	16	120	L	Digisonic SP ^N^
**S5**	F	65	20	Unknown	20	120	L	Digisonic SP ^N^
***Mean (std)***		***59*.*4 (8*.*3)***	***6*.*2*** ***(7*.*9)***		***16*.*0*** ***(6*.*4)***	***103*.*2*** ***(23*.*3)***		
**S6**	F	60	2	Sudden hearing loss	19	94	R	CI512 ^C^
**S7**	M	65	30	Sudden hearing loss	20	95	R	Digisonic SP ^N^
**S8**	F	54	3	Sudden hearing loss	20	66	R	CI512 ^C^
**S9**	M	65	10	Meniere’s	20	79	R	CI522 ^C^
***Mean (std)***		***61*.*0 (5*.*2)***	***11*.*3*** ***(13*.*0)***		***19*.*8*** ***(0*.*5)***	***83*.*5*** ***(13*.*8)***		

Dur deaf = duration of deafness; PTA = pure-tone average thresholds across 0.5, 1.0, 2.0, and 3.0 kHz

^C^ = Cochlear device

^N^ = Neurelec device

Eight normal hearing (NH) adults (6 women and 2 men) served as experimental controls. All had PTA thresholds ≤25 dB HL and none had any reported neuronal disease. The mean age at testing was 54±3.2 years (range: 50 to 60). All NH subjects had a mini mental state score of 30/30. Kruskal Wallis ANOVA showed no significant differences between the three groups (SSD-right-CI, SSD-left-CI and NH) in terms of age at testing (χ^2^ = 2.7, p = 0.260) or gender distribution (χ^2^ = 0.8, p = 0.700).

The Ethics Committee of the University Hospital of Tours specifically approved the protocol (N°ID RCB No 2015-A01249-40), and written informed consent was obtained from all subjects.

SSD-CI patients were tested before cochlear implantation (Base), and then 6 months (6m) and 12 months (12m) after CI activation. Speech understanding in noise and CAEPs were recorded at all three test sessions.

### Speech testing

**Fig 1:**
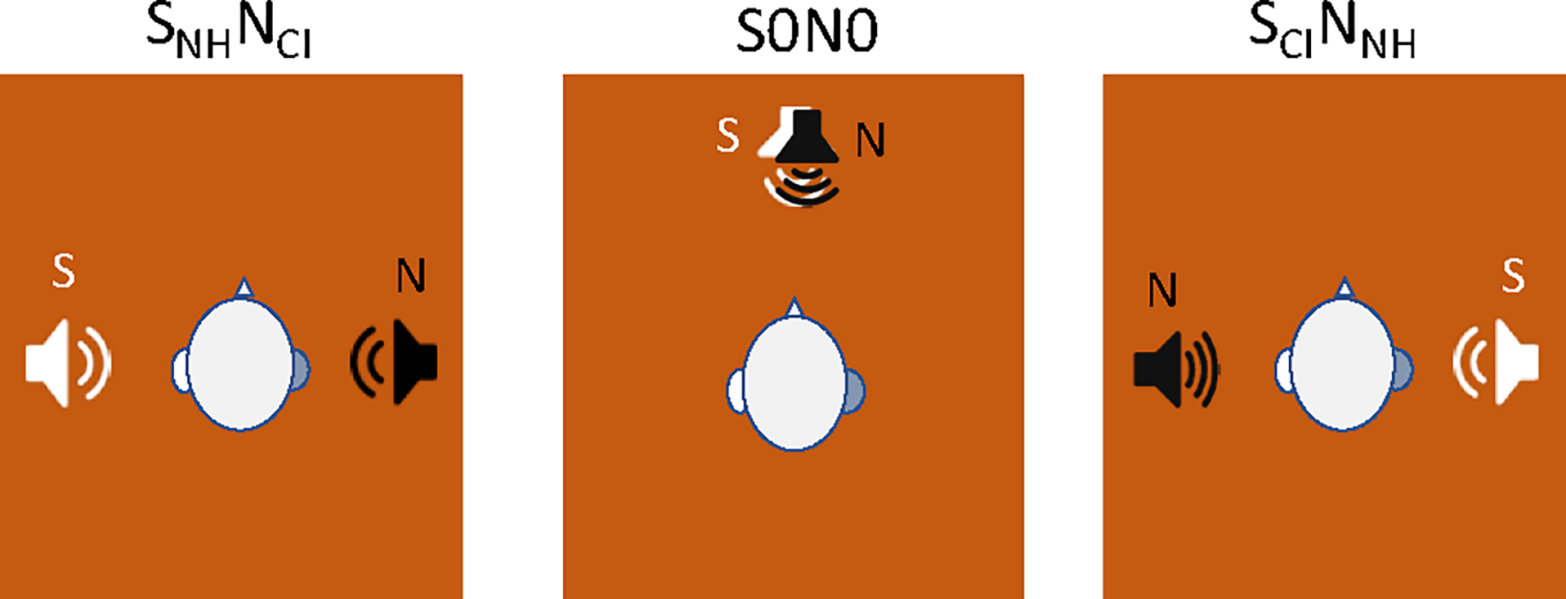
Spatial conditions for testing speech understanding in noise. Speech (S) was presented to the NH ear and noise (N) to the CI ear (S_NH_N_CI_), or S and N were co-located directly in front of the subject (S0N0), or S was presented to the CI ear and N to the NH ear (S_CI_N_NH_).

Sentence recognition in steady, speech-shaped noise was measured using an adaptive procedure. Stimuli consisted of French sentences from the Marginal Benefit from Acoustic Amplification (MBAA) corpus, which consists of 36 lists of 15 sentences each [45]. For each condition, a list was randomly selected (without replacement) and sentences within the list were randomly presented (without replacement) in sound field. Subjects were tested with binaural listening; after implantation, subjects were tested using their clinical processors and settings. Speech was presented at 65 dBA and the noise level was adjusted in 5 dB steps according to the correctness of the response. If the subject repeated the entire sentence correctly, the signal-to-noise ratio (SNR) was reduced by 5 dB. If the subject did not repeat the entire sentence correctly, SNR was increased by 5 dB. The final 6 reversals in SNR were averaged as the speech reception threshold (SRT), defined as the SNR required to produce 50% correct whole sentence recognition. Three spatial conditions were tested and are illustrated in Fig 1: 1) Speech to the NH ear, noise to the CI ear (S_NH_N_CI_), 2) Co-located speech and noise in front of the subject (S0N0), and 3) Speech to the CI ear, noise to the NH ear (S_CI_N_NH_).

### Cortical auditory evoked potentials (CAEPs)

#### Stimuli

The speech stimulus was /ba/ produced by a female voice and recorded in a soundproof booth. The fundamental frequency (F0) = 198 Hz, the first formant (F1) = 779 Hz, the second formant (F2) = 1369 Hz and the third formant (F3) = 2720 Hz. The duration of the stimulus was 125 ms. Stimuli (n = 1150) were presented at 70 dBA via 2 loudspeakers situated at 1.3 m away from the subject and -45° and +45° relative to center. Stimuli were presented with a constant inter-stimulus interval of 700 ms (offset to onset). The neurophysiological recordings lasted approximately 20 minutes per subject.

#### Electroecephalogram (EEG) data recording

During the recording session, subjects sat in a sound-attenuated room and watched a movie with the sound muted. EEG data were recorded using Compumedics System Neuroscan EEG system (Synamps RT amplifier and Curry 7 software) with 64 electrodes referenced on line to the nose; note that after cochlear implantation, only 61 of the 64 electrodes could be used due to the presence of the CI transmitter coil. All electrodes were placed according to the international 10–20 electrode placement standard. Electrode impedances were kept below 5 kΩ. In addition, electrooculogram (EOG) activity was recorded from electrodes placed at the outer canthi of both eyes (horizontal EOG) and above and below the right eye (vertical EOG). The EEG data were recorded with a sampling frequency of 500 Hz and low-pass filtered at 200 Hz. The stimulus presentation was controlled by Neuroscan Stim^2^ software.

**Fig 2:**
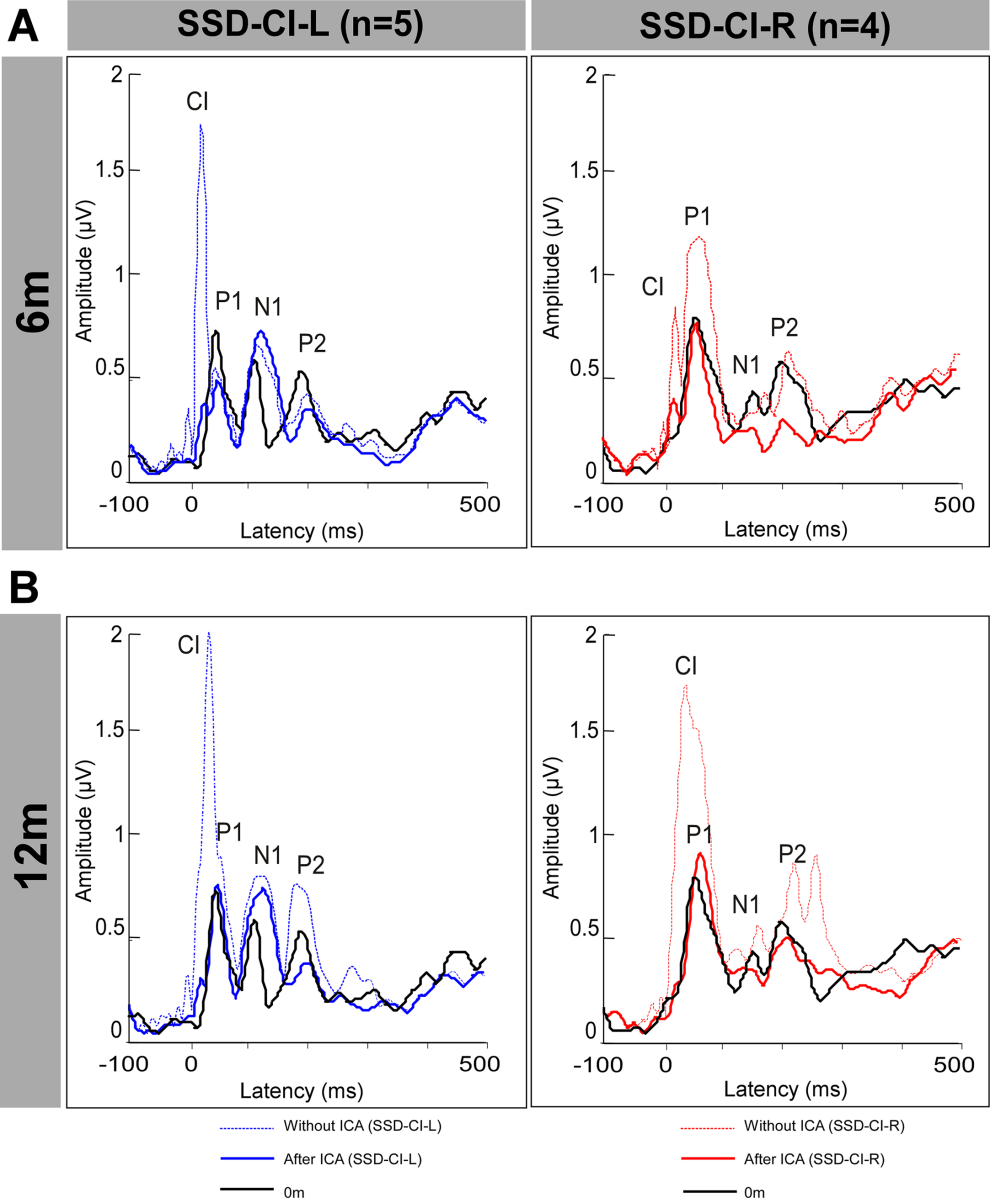
Mean GFP amplitude for SSD-CI left (n = 5) and right (n = 4) subjects, before and after ICA removal of CI artifact.

EEG analysis was performed using EEGLAB [46] running in the Matlab environment (Mathworks, Natick, MA). First, EEG recordings were filtered by a band-pass filter (0.3–70 Hz). EEG periods recorded during subject movement were identified visually and rejected; the mean artifact rejection was less than 25% per participant for test sessions. Extended infomax independent component analysis (ICA) implemented in EEGLAB was applied to the continuous data from each EEG to reduce CI-related artifact, as in Debener et al. (2008) [47]. ICA analysis assumes that the EEG data recorded at multiple scalp sensors are linear sums of temporally independent components arising from spatially fixed, distinct or overlapping brain sources. The unmixed data were decomposed into a sum of temporally independent and spatially fixed components. ICA components representing CI artifacts were identified by the centroid on the side of the implanted device time-locked to the auditory stimulation and had large amplitude. Independent components representing common EEG artifacts (e.g., eye blink and saccade) were visually identified and removed along with those components representing the CI artifacts. At 6m, an average of, 6.2 and 5 independent components were removed for SSD-CI-L and SSD-CI-R subjects, respectively. At 12 m, an average of 4.8 (SSD-CI-L) and 5.75 (SSD-CI-R) independent components were removed. Fig 2 shows global field power (GFP) amplitude for the mean of the 5 SSD-CI-L and for the mean of the 4 SSD-CI-R subjects, with and without ICA artifact removal. ICA removed substantial amount of artifact, especially for the SSD-CI-L subject. For both subjects, ICA removed the CI artifact while preserving P1 amplitude. Afterwards, EEG was segmented into epochs from -100 to 500 ms relative to the stimulus onset and re referencing to average reference. The epochs were baseline-corrected relative to a 100-ms pre-stimulus time window, and a digital zero-phase-shift low-pass filter of 30 Hz was applied. The mean number of epochs varied from 748 at Base, 693 at 6m, and 692 at 12m for the SSD subjects and 746 for NH subjects.

CAEP analysis were performed with ELAN software [48], and scalp potential maps were created from the CAEP data [49]. Mean averaged waveform for each eliciting stimulus were obtained separately for each subject. Data from missing electrodes due to the CI were interpolated. Scalp potential maps were generated using a two-dimensional spherical spline interpolation [49] and a radial projection from Cz (top views), T3 (left lateral view) or T4 (right lateral view), with respect to the length of the meridian arcs.

### Statistical analysis

#### Behavioral analyses

When there was a normal distribution of data, parametric repeated measures analyses of variance (RM ANOVAs) were performed. When the distribution of data was not normal, non-parametric Friedman or Man-Whitney tests were used.

#### CAEP analysis

GFP analysis provides a reference-free measure for component identification that is based on the spatial root mean square (RMS) voltage deviations across electrode recordings from the entire scalp [50–52]. GFP allows for a global measure of the electric field at the scalp. This analysis type avoids the bias of the experimenter’s selection of a single electrode (or set of electrodes) for identifying the components within the epoch used to visualize the waveform. The effect of test session (Base, 6m, 12m) on GFP peak amplitude and latency for CAEP components P1, N1 and P2.

To evaluate evolution at the side contralateral to CI ear, CAEP analysis was performed on sites M1, T7 and P7 for SSD-CI-R, and M2, T8 and P8 for SSD-CI-L. Due to CI artifact, CAEPs were not analyzed for the sites ipsilateral to the CI ear. For frontocentral areas, P1, N1 and P2 amplitude and latency were analyzed for P1, N1 and P2. The T-complex components (Na, Ta, Tb) at temporal sites and the reversal polarity at mastoid sites [P1(RP), N1(RP) and P2(RP)] were investigated by visual inspection from baseline to peak for each subject. Peak identification for GFP and CAEP components were reviewed by a second investigator to check the consistency of the data with an inter-judge agreement rate of 95%. The two investigators found a consensus for the remaining 5%.

A multivariate analysis of covariance (MANCOVA) was used, with test session, group and CAEP components as fixed factors, amplitude and latency as dependent variables, and subject as the co-varying factor. The significance level was p<0.05 and post-hoc Bonferroni pairwise comparisons were performed for significant effects and/or interactions. Data from NH listeners were compared to SSD-CI-L and SSD-CI-R data at each test session using non-parametric Mann-Whitney tests; Bonferroni correction was applied for multiple comparisons and the adjusted significance level was p<0.017. Spearman correlations were used to compare speech perception scores to GFP amplitude and latency, CAEP component amplitude and latencies, and duration of deafness.

## Results

### Speech understanding in noise

**Fig 3:**
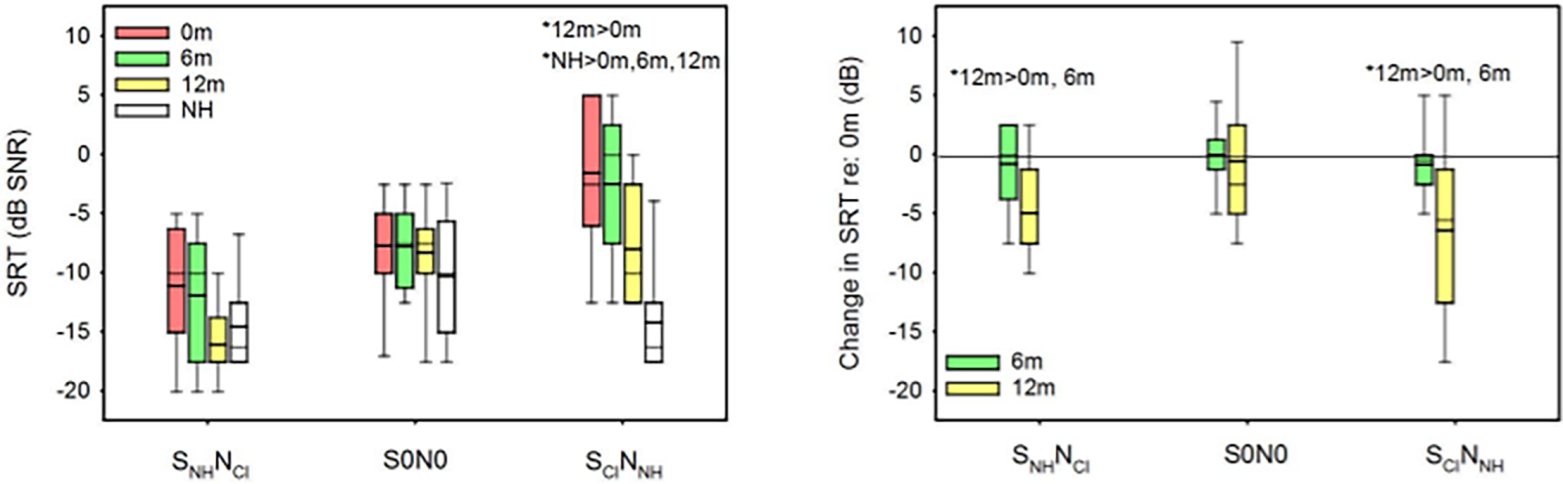
SRTs measured for the three spatial conditions. A: Mean SRTs for different spatial conditions. The colored bars show SSD-CI performance at Base (red), 6m (green), and 12m (yellow). The white bars show performance for the NH control group. B: Mean change in SRTs for SSD CI subjects, relative to baseline; values <0 indicate improved speech understanding in noise, relative to baseline. In both panels, the boxes show the 25^th^ and 75^th^ percentiles, the error bars show the 5^th^ and 95^th^ percentiles, the thick line shows the median, and the thin line shows the mean. The asterisks show significant differences between test sessions or between subject group (p<0.05).

Fig 3A shows SRTs in noise for the SSD CI subjects for the 3 spatial conditions, as a function of test sessions; NH data is also shown for each spatial condition. Fig 3B shows the change in SRT for CI patients after implantation relative to baseline. For S_NH_N_CI_, the mean SRT for SSD CI subjects was reduced by 0.8 dB at 6m and by 3.9 dB at 12m, relative to baseline; the mean SRT decreased by 3.1 dB between 6m and 12m. For S0N0, the mean SRT was reduced by 0.1 dB at 6m, and by -0.6 dB at 12m, relative to baseline; the mean SRT decreased by 0.6 dB between 6m and 12m. For S_CI_N_NH_, the mean SRT was reduced by 0.9 dB at 6m, and by 7.6 dB at 12m, relative to baseline; the mean SRT decreased by 6.7 dB between 6m and 12m.

Among SSD-CI subjects, a two-way RM ANOVA, with CI side (left, right) and test session (Base, 6m, 12m) as factors, showed a significant effect of test session for S_NH_N_CI_ [F(2,14) = 9.5, p = 0.002] but not for CI side [F(1,14) = 0.8, p = 0.386]; there was no significant interaction [F(2,14) = 1.8, p = 0.210]. For S0N0, there was no significant effect of test session [F(2,14) = 0.1, p = 0.965] or CI side [F(1,14) = 0.3, p = 0.613]. For S_CI_N_NH_, there was a significant effect of test session [F(2,14) = 6.2, p = 0.012], but not for CI side [F(1,14) = 0.1, p = 0.809]; there was no significant interaction [F(2,14) = 0.4, p = 0.666]. A Mann-Whitney test showed no significant difference in SRTs NH subjects and SSD-CI subjects at any test session for S_NH_N_CI_ and S0N0 (p>0.05 in all cases). For S_CI_N_NH_, NH performance was significantly better than SSD-CI performance at all test sessions (p<0.05 in all cases).

### Evolution of cortical responses for SSD CI subjects

#### GFP analysis

**Fig 4:**
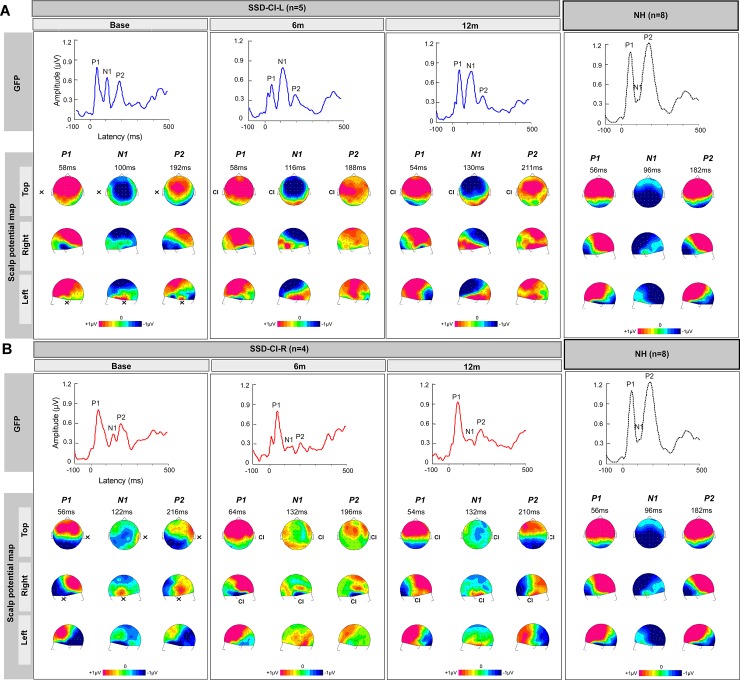
Evoked surface activity over time of CI stimulation (Base, 6m, 12m) for SSD-CI subjects and NH control group. (A) Top: Mean GFP for SSD-CI-L subjects (n = 5); NH GFP is shown at right. Bottom: mean scalp potential maps (right, top and left views) for P1, N1 and P2 peak latency; NH maps are shown at right. (B) Top: Mean GFP for SSD-CI-R subjects (n = 4); NH GFP is shown at right. Bottom: mean scalp potential maps (right, top and left views) for P1, N1 and P2 peak latency; NH maps are shown at right.

Three peaks were observed for GFP in SSD-CI-L (Fig 4A) and SSD-CI-R subjects (Fig 4B): P1, N1 and P2. Mean amplitude and latencies across groups are shown in S1 Fig. For SSD-CI-L patients, mean P1 and N1 peak amplitude appeared to increase across test sessions, with no change in latency (Fig 4A and S1 Fig). For SSD-CI-R patients, there did not appear to be any increase in mean peak amplitude or latency across test sessions (Fig 4B and S1 Fig); indeed, mean N1 and P2 peak amplitude appeared to decrease at 6m. The effects of test session (Base, 6m, 12m), group (SDD-CI-L, SSD-CI-R) and CAEP (P1, N1, P2) were evaluated using MANCOVA; amplitude and latency were the dependent variables and subject was the co-varying factor. Complete statistical results are shown in S1 Table. No significant effects of test session, group and peaks were observed for GFP (p>0.05 in all cases).

#### CAEP analysis

**Fig 5:**
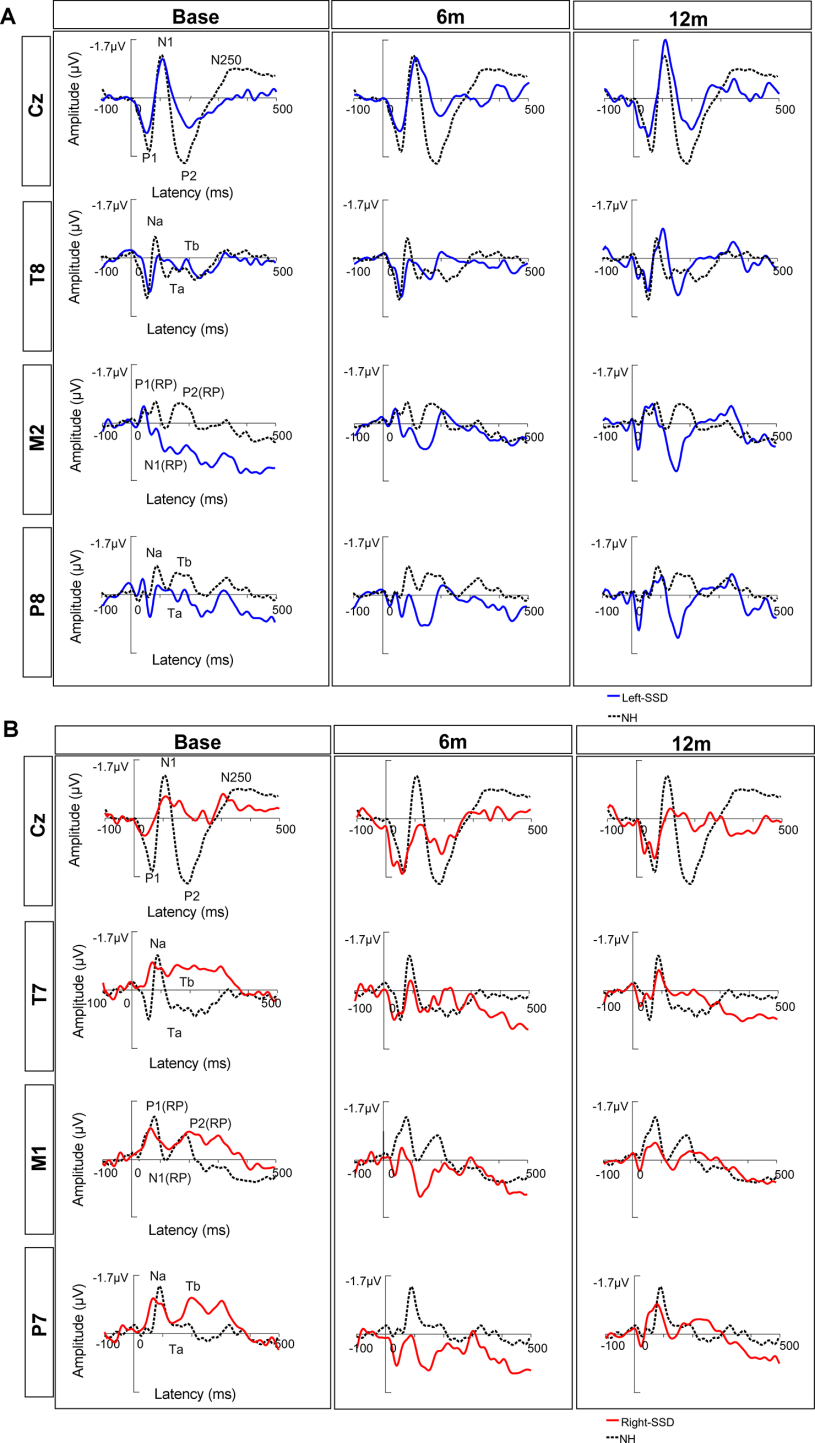
**Mean CAEPs recorded at different test sessions (Base, 6m, 12m) for SSD-CI-L (A) and SSD-CI-R patients (B), and for the NH control group**. SSD-CI-L data are shown in blue, SSD-CI-R data are shown in red, and NH data are shown in black (dotted line).

Fig 5 shows CAEPs measured at frontocentral (Cz), temporal and mastoid sites for SSD-CI patients and NH listeners. For SSD-CI-L patients, temporal sites T8 and P8 and mastoid site M2 were used (i.e., contralateral to the implanted ear); for SSD-CI-R patients, temporal sites T7 and P7 and mastoid site M1 were used. Mean peak amplitude is shown in S2 Fig, and mean peak latency is shown in S3 Fig. As with the GFP data, CAEP data were analyzed using MANCOVA, with test session, group and CAEP as fixed factors, amplitude and latency as dependent variables, and subject as the co-varying factor; complete results for all sites are shown in S1 Table.

At Cz, there did not appear to be much change in P1, N1, or P2 amplitude or latency across test sessions for SSD-CI-L patients. There were no consistent changes in amplitude or latency for SSD-CI-R patients after implantation, relative to baseline. The MANCOVA showed no significant effects of test session or group, and no interactions among fixed factors (p>0.05).

At temporal and mastoid sites, CAEP morphology in the contralateral ear to the CI ear appeared to change across test session for both CI groups. At Base, the different peaks were not easy to identify, but grew in amplitude at 12m. This was more pronounced for SSD-CI–L (Fig 5A) than for SSD-CI-R patients (Fig 5B).

The MANCOVA performed at temporal sites T7 (SSD-CI-R) and T8 (SSD-CI-L) showed no significant effects for time or group (p>0.05); complete results are shown in S1 Table. However, there was a significant interaction between time and group for Na latency at 12m (p = 0.028), with longer latency for SSD-CI-L than SSD-CI-R patients. No other interactions were found (p>0.05). For P7 (SSD-CI-R) and P8 (SSD-CI-L), there was a significant group effect for amplitude (p = 0.047), but no significant effect of time (p>0.05), and no significant interactions between remaining factors.

The MANCOVA (complete results in S1 Table) performed at mastoid sites M1 (SSD-CI-R) and M2 (SSD-CI-L) showed significant effects of time for amplitude (p = 0.001) and group for amplitude (p = 0.001) and latency (p<0.001). Significant interactions were observed between time and CAEP for amplitude (p = 0.010) and between group and CAEP for latency (p<0.001). Post-hoc Bonferroni pairwise comparisons showed that amplitude was significantly higher at 6m and 12 m, relative to Base (p<0.05 in both cases). Amplitude was significantly higher for SSD-CI-L than for SSD-CI-R patients for P1(RP) at Base and 6m (p<0.05), for N1(RP) at Base and 12 m (p<0.05) and for P2(RP) at Base (p<0.05). Latency was significantly longer for SSD-CI-L than for SSD-CI-R patients for P1(RP) and N1(RP) (p<0.05 in both cases). For N1(RP), amplitude was significantly higher at 6m and 12 m than at Base (p<0.05 in both cases), and latency was significantly longer for SSD-CI-L than for SSD-CI-R patients.

#### Topographic evolution across time for SSD CI subjects

Scalp potential maps for P1, N1 and P2 peaks were generated at Base, 6m, and 12m for SSD-CI-L and (Fig 4A) SSD-CI-R subjects (Fig 4B). For SSD-CI-L patients, at P1 and P2 peak latencies, the scalp potential maps did not appear to change across test session and presented a positive activity at frontocentral areas. For N1, an asymmetric distribution of the positive field at masto-temporal areas was observed at Base; at 6m and 12m, a bilateral positive field at was observed. Also, the negative field at frontocentral electrodes was more pronounced at 6m and 12m.

For SSD-CI-R subjects, at P1 and P2 peak latencies, the scalp potential maps exhibited a positive field at frontocentral areas, which did not appear to change across test sessions. For N1, there was a very low negative frontocentral activity at Base, 6m and 12m. At temporal sites, only the side ipsilateral to the CI ear exhibited a large positive activity at Base and 12m. For the side contralateral to CI ear, the temporal activity was negative at Base and positive at 12m.

### Comparison between SSD and NH groups

#### GFP analysis

The GFP shown in Fig 4 showed a higher P2 peak amplitude for the NH group than for SSD-CI patients and a lower N1 amplitude than for SSD-CI-L patients. The GFP amplitude and latency at each peak were compared between NH subjects and SSD-CI subjects at each test session using Kruskal-Wallis tests; complete results are shown in S2 Table and mean amplitudes and latencies are shown in S1 Fig. After Bonferroni correction for multiple comparisons (adjusted p<0.017), no significant difference was observed between NH and SSD-CI patients for amplitude. Latency for P2 at 12m was significantly longer for SSD-CI-L (p = 0.015) and SSD-CI-R patients (p = 0.008).

#### CAEP analysis

The CAEP morphology is presented in Fig 5 and mean peak amplitudes and latencies are shown in S2 Fig and S3 Fig, respectively. The CAEP amplitude and latency at each peak were compared between NH subjects and SSD-CI subjects at each test session using Kruskal-Wallis tests; complete results are shown in S2 Table.

At Cz, there was no significant difference in P1, N1, or P2 amplitude or latency between NH and SSD-CI-L subjects at all test sessions. For P2, NH amplitude was significantly higher for NH than for SSD-CI-R subjects (p = 0.008). For N1, latency was significantly longer for SSD-CI-R subjects at 6m than for NH subjects (p = 0.008).

At temporal sites (T7, T8; P7, P8), there was no significant difference in peak amplitude between NH and SSD-CI subjects. There was also no significant difference in peak latency between NH and SSD-CI subjects, except for Tb at P8, where latency was significantly longer for NH subjects than for SSD-CI-L subjects at 6m (p = 0.010).

At mastoid sites (M1, M2), there was no significant difference in peak amplitude between NH and SSD-CI subjects. There was also no significant difference in peak latency between NH and SSD-CI subjects, except for N1(RP) at M2, where latency was significantly shorter for NH subjects than for SSD-CI-L subjects at 12m (p = 0.002).

#### Topographic comparison between NH group and SSD-CI

As shown in Fig 4, scalp potential maps for the NH group showed a high positive P1 peak amplitude at frontocentral areas, similar to SSD-CI patients at all sessions. Scalp potential maps also showed that the NH group exhibited a high negative N1 peak amplitude at frontocentral and posterior areas, with amplitudes close to zero at mastoid and temporal sites. SSD-CI-L patients similarly exhibited negative N1 amplitude at frontocentral areas; the pattern was different for SSD-CI-R patients, who exhibited a lower negative N1 wave amplitude and a more diffuse pattern of activation, mostly at 6m. The positive field observed for the NH group at temporal and mastoid sites appeared to be lower than that of SSD-CI-L patients at 12m and 6m, but was closer to that of SSD-CI-R at 12m. For P2, the scalp potential maps showed higher amplitude for the NH group than for SSD-CI patients at each session. This difference was even more pronounced for SSD-CI-R patients at Base and 6m, where frontocentral activity was close to zero; at 12m, frontocentral activity was closer to NH group. Frontocentral activity was more similar between SSD-CI-L patients and the NH group.

### Correlations

Speech understanding in noise was compared to GFP amplitude and latency at each test session. Spearman correlations showed no significant relationship between speech performance and GFP amplitude or latency at any test sessions for any of the spatial conditions (p>0.05 in all cases). A similar lack of correlation was observed between speech performance and CAEP components values at mastoid-temporal sites (p>0.05 in all cases). Speech performance was also compared to duration of deafness; a significant correlation was observed for S_CI_N_NH_ at 12m (r = 0.67, p = 0.047), with speech performance worsening with increasing duration of deafness.

## Discussion

This longitudinal study provides evidence that restoration of binaural function with a CI in adult SSD patients can induce changes in in GFP and in cortical responses to auditory stimuli on the hemispheric side contralateral to the implanted ear for SSD-CI users.

### Changes in CAEPs after cochlear implantation

Cortical modification seemed to occur rapidly in the (admittedly small) group of SSD-CI-L subjects, with an increase in N1 amplitude for GFP (Fig 4A) at 6m and at Cz (Fig 5A) at 12m, relative to baseline. In contrast, SSD-CI-R subjects exhibited no increase in N1 amplitude for GFP (Fig 4B) or Cz (Fig 5B) after cochlear implantation. The scalp potential maps for the ear contralateral to the CI (Fig 4) showed a substantial increase in positivity between Base and 12m, suggesting activation of contralateral temporal generators [52]. This was also supported by observation of the waveforms from electrodes situated at the side contralateral to the CI ear (Fig 5), with more identifiable peaks at 12m relative to Base. However, the MANCOVA analyses showed no significant changes in CAEP amplitude for GFP, Cz or temporal sites. A significant effect of test session was only observed at mastoid sites, with N1(RP) amplitude significantly increasing at 6m and 12m, relative to Base. The lack of significant increases in N1 amplitude for GFP, Cz and temporal sites was most likely due to the limited number of subjects, resulting in low statistical power. Note that P1 and P2 amplitudes did not significantly change over time for GFP, Cz, or for the scalp potential maps. For P1, this might reflect an absence of change at the thalamocortical level [25]. For P2, this might reflect no change in the mesencephalic reticular activating system [25,53,54].

Previous and present data suggest some cortical reorganization after reactivating the temporal areas via CI stimulation. Such cortical reorganization is consistent with previous studies for patients with a profound bilateral hearing loss after cochlear implantation [47,55]. Previous studies found comparable results in adults after otosclerosis surgery who exhibited increased contralateral auditory cortex responses after hearing recovery, with the extent of activated cortex becoming bilateral at 9 months post-surgery and including a greater portion of the posterior superior temporal plane [17]. Other studies have reported similar effects on cortical auditory pathways pediatric SSD patients after cochlear implantation [42–44], where children revealed remarkable developmental plasticity within a 6-month period. Polonenko et al. [44] studied young SSD-CI-R children and found an atypical distribution of peak amplitude activity from the implanted ear after CI activation, marked by an abnormal lateralization of activity to the ipsilateral left auditory cortex. After chronic implant use, a contralateral aural preference emerged in both auditory cortices. Here, adult SSD-CI-R subjects presented an atypical distribution at temporal sites for N1 peak at 6m, with a negative field on scalp potential map on the side ipsilateral to CI ear and a very low positive field at the ear contralateral to CI ear (Fig 4). At 12m, increased activation of the side contralateral to CI was observed. This suggests a similar development of cortical reorganization between young and adult SSD-CI-R patients.

Other studies with unilaterally deaf adults found that cortical reorganization mainly occurred for patients who were deaf in the left ear [56,57]. Here, cortical reorganization after binaural recovery was more pronounced for SSD-CI-L patients, who exhibited higher N1 peak and N1(RP) amplitudes at 12m for GFP, Cz and mastoid sites than did SSD-CI-R patients. The Ta peak at temporal sites also appeared to have a higher amplitude for SSD-CI-L patients. This suggests a faster reorganization for SSD CI patients implanted on the left side. On another hand, there was no significant effect of test session on peak amplitude for GFP or at Cz. This hypothesis was supported by a significantly higher amplitude for SSD-CI-L patients for Na at temporal sites T8 and P8 at 6m, mastoid site M2 for P1(RP) at 6m, N1(RP) at 12m. Significantly longer latency was also observed for SSD-CI-L at mastoid site M2 for N1(RP) and P2(RP), and for temporal site T8 for Na. This suggests that there may be a later distribution of peak activity in SSD-CI-L patients for some components [58].

### NH versus SSD-CI cortical responses

For N1 peak latency at all sessions, SSD-CI-L patients exhibited greater differences between temporal (positive field) and frontocentral (negative field) areas than did SSD-CI-R patients or the NH group (scalp potential maps in Fig 4). The greater contrast among electrodes results in a higher standard deviation, resulting in a larger N1 amplitude for GFP [58]. Previous studies that explored CAEP in response to voice stimuli generally found low N1 amplitude for NH adults [24,59]. This may explain the low N1 peak amplitude for GFP in the NH group. Note that the N1 amplitude for SSD-CI-L subjects was not significantly higher than that for SSD-R-CI or NH subjects; again, the low statistical power may have resulted in the non-significant finding. As shown in Fig 5 and S2 Fig, SSD-CI-L appeared to exhibit higher peak amplitudes at 12m for the M2, P8 and T8 sites than did the NH group. The higher N1 amplitude at 12m might be due to cortical reorganization, which would induce more neuronal elements activated synchronously than in the NH group. The GFP was more comparable between SSD-CI-R patients and the NH group. It is possible that cortical reorganization and mechanisms may be different from left- and right-implanted SSD patients.

For GFP and at Cz site, P1 and P2 peak amplitude were generally higher for the NH group than for SSD CI patients, especially for P2 peak. This might indicate differences in the neural processes that generate P1 and P2 waves between NH and SSD patients, even after cochlear implantation. These differences are further supported by the lack of significance change in peak amplitude after cochlear implantation for GFP and Cz site for SSD-CI-L patients, with significantly lower amplitude observed only for SSD-CI-R patients at baseline for P2.

The GFP latency was significantly shorter for the NH group than for SSD-CI-L subjects for P2 at 12m, and significantly shorter for the NH group than for SSD-CI-R subjects for N1 at 12m. A significant longer latency was observed for SSD-CI-L subjects than for the NH group at some temporal (Tb at P8 for SSD-CI-L at 6m), mastoid [N1(RP) at M2 for SSD-CI-L at 12m] and frontocentral sites (N1 at Cz for SSD-CI-R at baseline). As noted above, this suggests that the maximal activity in the potential distribution may occur later in SSD patients for some components, even after cochlear implantation [58].

### Speech understanding in noise improves with CI experience

No significant changes in SRTs were observed for the S_NH_N_CI_ or the S0N0 conditions over the one-year period, consistent with previous studies [16,18,60–62]. Most likely, the non-implanted ear dominated binaural perception such that there was no significant difference between NH and SSD-CI performance. For S_CI_N_NH_, SRTs were significantly better after one year of experience with CI. All SSD CI subjects exhibited better performance in the S_CI_N_NH_ condition, and all used their CI for the majority of daily listening (data logging showed a mean of 12 hours of CI usage per day). This underscores the importance of wearing the CI as much as possible to maximize its benefit. However, SSD-CI performance for S_CI_N_NH_ remained significantly poorer than that of NH listeners, even after one year of CI experience.

In this study with SSD-CI patients, cochlear implantation was associated with improved speech understanding in noise, increased GFP amplitude for N1 peak for SSD-CI-L patients, and a positive field at temporal sites contralateral to CI ear on scalp potential map for all SSD-CI patients. However, SSD behavioral outcomes were highly variable and may depend on many factors, including the degree, laterality and duration of hearing loss [1,56,57]. Duration of deafness has been shown to significantly affect adult CI outcomes in bilaterally deaf patients [63]. The negative effects of long-term deafness may arise from deprivation and/or non-use of the auditory cortex [64]. This underlines the importance of cochlear implantation for SSD patients as soon as possible to take advantage of neural plasticity.

In this study, there was a significant correlation between duration of deafness and SRTs for the S_CI_N_NH_ condition at 12m. Thus, for some SSD-CI subjects, 12 months of CI experience was not sufficient to overcome the longer period of auditory deprivation. In adult humans, previous studies have shown that auditory plasticity can be observed within the first weeks after onset of unilateral deafness [10,65]. Ponton et al. [8] suggested that changes in cortical activity occur gradually and may continue for 2 years or more after the onset of hearing loss. This suggests that auditory plasticity may interact with the duration of deafness, underscoring the need to implant patients with unilateral deafness sooner rather than later.

### Limits and caveats

Recording cortical responses with EEG can be difficult due to the CI artifact related to CI device, the mode of stimulation and the surgical placement of the remote return electrode [47,66,67]. The reduction of the CI artifact with ICA allowed for the study of scalp potential distribution and analysis of the waves. However, this method cannot determine whether the artifact rejection significantly distorts the measured cortical responses, as is possible with other methods [68]. As shown in Fig 2, ICA appeared to successfully remove CI artifact while preserving key aspects of the cortical response.

In this study, auditory stimuli were delivered in sound field via two loudspeakers positioned +45° and -45° from center. This did not allow for selective delivery of stimuli to each ear (e.g., with insert earphone to the normal-hearing ear and direct audio input to the CI ear). This presentation setup was used to mimic the everyday listening conditions for SSD-CI patients, and because it is difficult to control the relative loudness levels between acoustic and electric stimulation. Indeed, responses from the CI side may be influenced by speech processor settings such as microphone gain, volume level, acoustic-to-electric amplitude mapping, etc. Although SSD-CI subjects were tested with their clinical processors and settings, the electrode dynamic range may have been different at 6m and 12m. As such, it was difficult to analyze hemispheric asymmetries in this study. Another approach would be to record responses at 6m or 12m with the CI off to observe any changes in responses relative to Base. Unfortunately, this was not done due to time constraints.

Previous animal studies have investigated cortical reorganization for acquired or congenital SSD. Unilateral deafness produces profound changes in the cortical structure and in the central auditory system, thus modifying the cortical asymmetry in the pattern of auditory cortex responses [69]. Studies with congenital or early-onset unilateral deafness in cats show a neuronal reorganization in terms of aural preference for the NH ear, which is different for acquired deafness [70]. Different from acquired hearing loss, congenital unilateral hearing loss results in a rearrangement of the binaural connections in the auditory brainstem, as well as changes in the physiology of inferior colliculus neurons [1,71,72]. In this study, all SSD-CI subjects had acquired hearing loss, and we cannot speculate on cortical reorganization for SSD patients with congenital deafness. However, Wedekind et al. [73] found that duration of deafness in the implanted ear had no impact on cortical reorganization in four SSD patients with early onset of deafness.

While this study showed that some cortical auditory reorganization occurred during the first year of CI experience, the number of SSD subjects was limited, with only 5 subjects implanted on the left side and 4 on the right side. Additional longitudinal studies should be conducted with more SSD-CI patients to better understand when cortical reorganization begins, how it may differ between left- and right-implanted subjects, and how long it persists.

## Conclusion

In this longitudinal study, speech understanding in noise and cortical responses were measured in SSD patients before cochlear implantation and at 6 and 12 months after implantation, and in a control group of NH subjects. Results showed that speech understanding in noise (S_CI_N_NH_) significantly improved after 12 months of experience with the CI. GFP significantly increased for SSD-CI-L subjects at N1 peak. Bilateral activation of temporal generators appears after cochlear implantations for both types of SSD, which could testify of a cortical reorganization.

## Supporting information

S1 Fig Mean peak amplitudes and latencies for GFP for SSD-CI-L (left panels), SSD-CI-R (right panels) and NH subjects (all panels); the error bars show the standard deviation.
(TIF)

S2 Fig Mean peak amplitudes for SSD-CI- L (left panels; Cz, T8, P8 and M2) and SSD-CI- R (right panels; Cz, T7, P7 and M1), and NH subjects (all panels); the error bars show the standard deviation.
(TIF)

S3 Fig Mean peak latencies for SSD-CI- L (left panels; Cz, T8, P8 and M2) and SSD-CI- R (right panels; Cz, T7, P7 and M1), and NH subjects (all panels); the error bars show the standard deviation.
(TIF)

S1 Table Results of MANCOVAs for GFP, Cz, temporal (P7, P8; T7, T8) and mastoid sites (M1, M2). Sites were selected to be contralateral to the implanted ear. For Test session (Base, 6m, 12m), group (right- or left-implanted), CAEP components were fixed factors, amplitude (Amp) and latency (Lat) were the dependent variables), and subjects were the co-varying factor. The asterisks and italics indicate significant effects.
(DOCX)

S2 Table Results of Mann-Whitney tests comparing GFP and CAEP amplitude and latency at each test sessions between NH and SSD-CI subjects. Italics indicate significant effects after Bonferroni adjustment for multiple comparisons (p<0.017).
(DOCX)

S1 Data Supplemental data. (XLSX)
