# Supplementary figures and images for: Cortical reorganization after cochlear implantation for adults with single-sided deafness
Source: PLoS One. 2018 Sep 24;13(9):e0204402. doi: 10.1371/journal.pone.0204402 (PMC6152998; doi:10.1371/journal.pone.0204402)

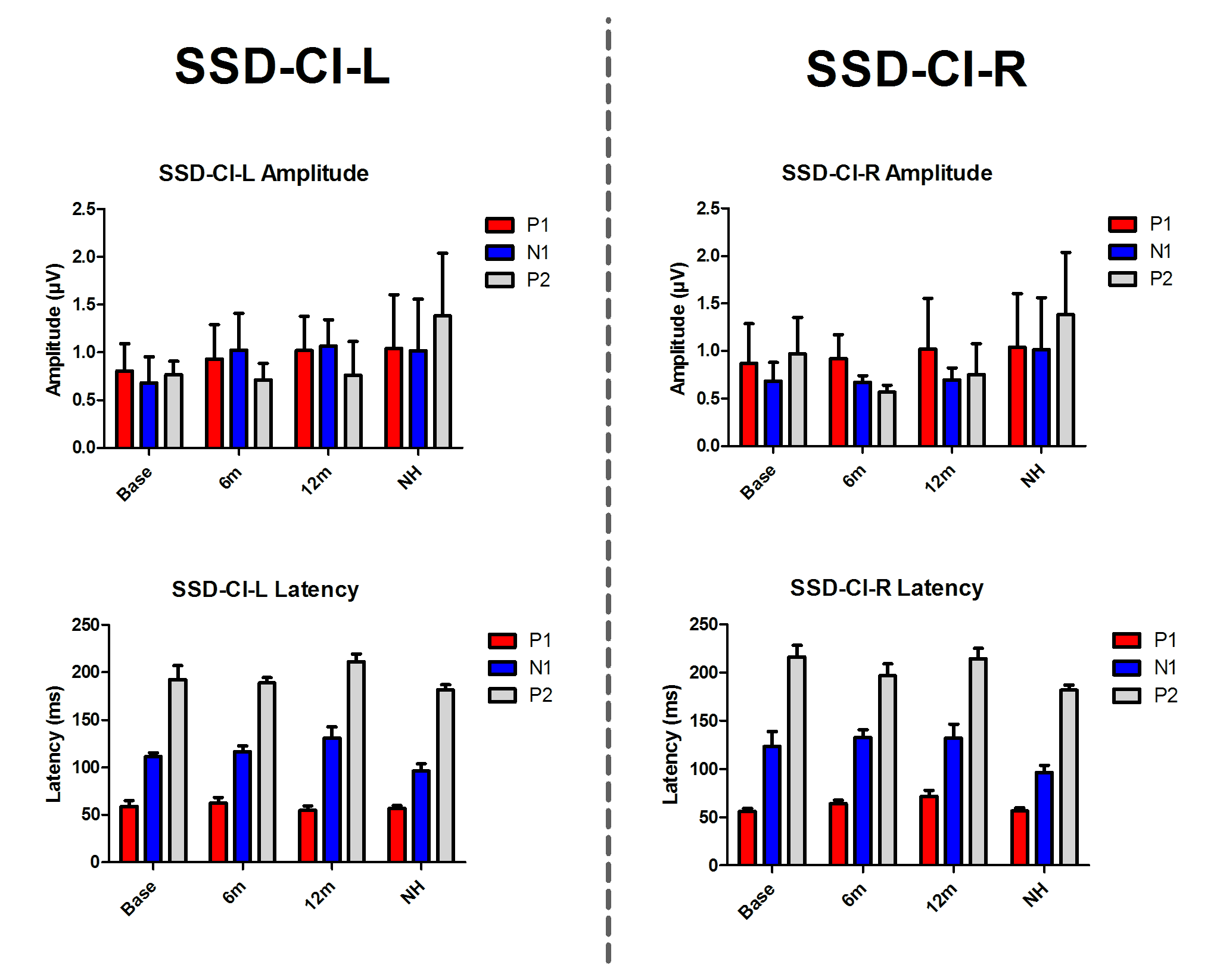

Supplement: S1 Fig — Mean peak amplitudes and latencies for GFP for SSD-CI-L (left panels), SSD-CI-R (right panels) and NH subjects (all panels); the error bars show the standard deviation. (TIF) [file pone.0204402.s001.tif]

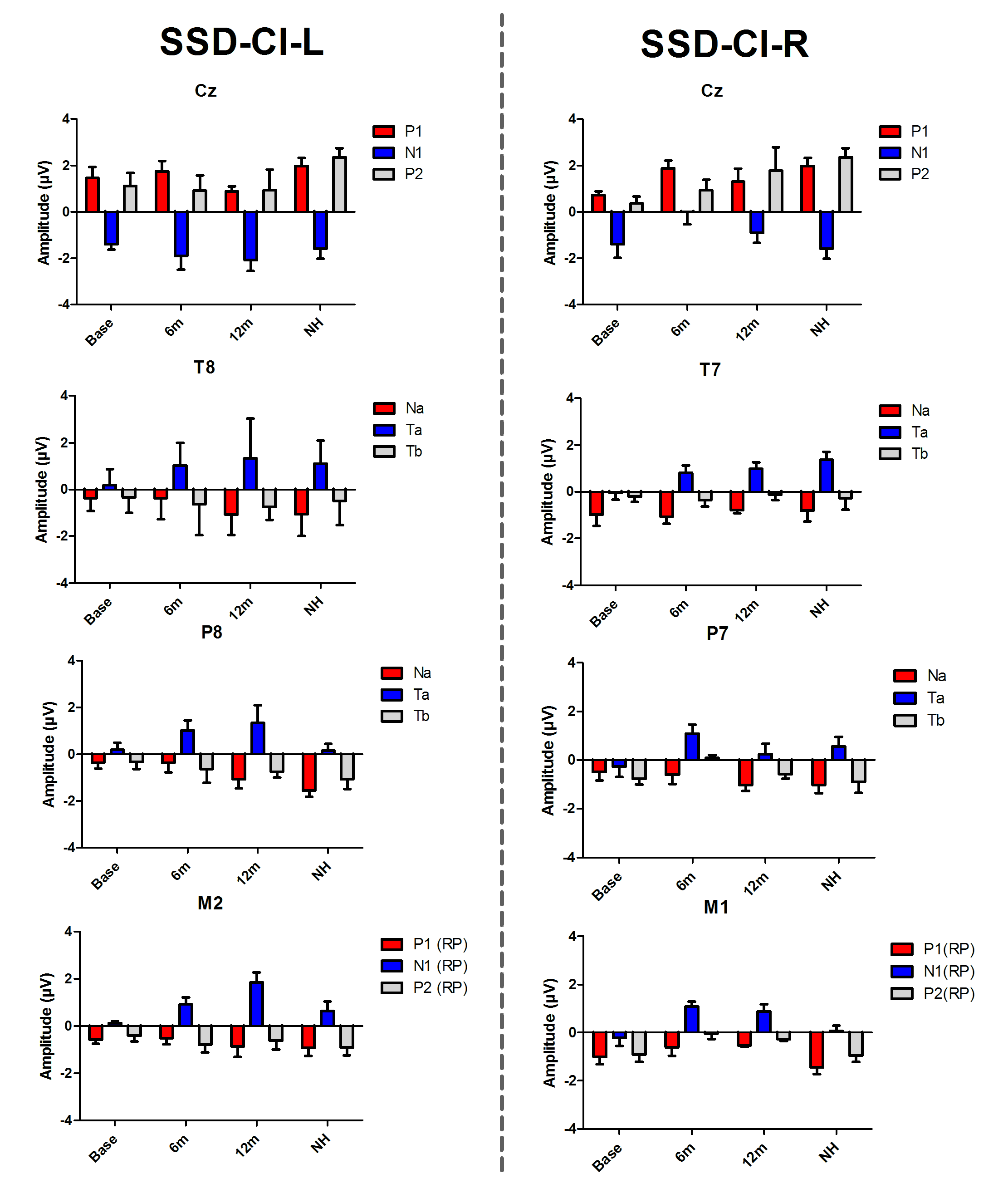

Supplement: S2 Fig — Mean peak amplitudes for SSD-CI- L (left panels; Cz, T8, P8 and M2) and SSD-CI- R (right panels; Cz, T7, P7 and M1), and NH subjects (all panels); the error bars show the standard deviation. (TIF) [file pone.0204402.s002.tif]

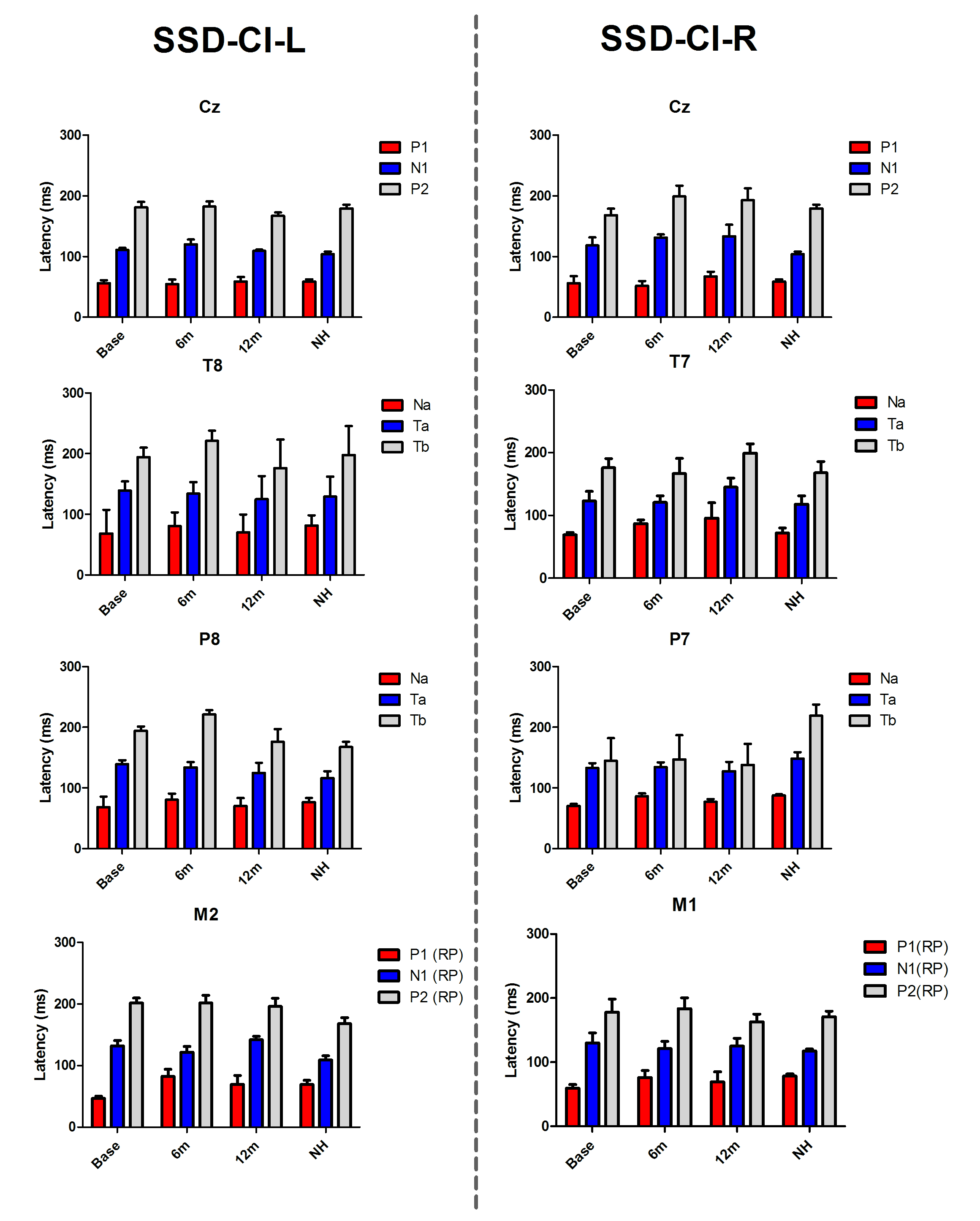

Supplement: S3 Fig — Mean peak latencies for SSD-CI- L (left panels; Cz, T8, P8 and M2) and SSD-CI- R (right panels; Cz, T7, P7 and M1), and NH subjects (all panels); the error bars show the standard deviation. (TIF) [file pone.0204402.s003.tif]
