# Supplementary material for: Cortical reorganization after cochlear implantation for adults with single-sided deafness
Source: PLoS One. 2018 Sep 24;13(9):e0204402. doi: 10.1371/journal.pone.0204402 (PMC6152998; doi:10.1371/journal.pone.0204402)
Supplement: S1 Table — Sites were selected to be contralateral to the implanted ear. For Test session (Base, 6m, 12m), group (right- or left-implanted), CAEP components were fixed factors, amplitude (Amp) and latency (Lat) were the dependent variables), and subjects were the co-varying factor. The asterisks and italics indicate significant effects. (DOCX) [file pone.0204402.s004.docx]

**S1 Table: Results of MANCOVAs for GFP, Cz, Mastoid (M1, M2) and Temporal (P7, P8; T7, T8) sites.** Amplitude (Amp) and latency (Lat) were analyzed according time, group, CAEP components (CAEP) factors and interactions. The asterisks and italics indicate significant effects. For SSD-CI-L subjects, M2, P8, and T8 sites were used (i.e., contralateral to the implanted ear); for SSD-CI-R subjects, M1, P7, and T7 sites were used.

|  |  |  | **df** | **F** | **Sig.** | **ŋ^2^** | **Observed power** | **Post-hoc (p<0.05)** |
| --- | --- | --- | --- | --- | --- | --- | --- | --- |
| **GFP** | CAEP | Amp | 2 | 2.3 | 0.108 | 0.07 | 0.45 |  |
|  |  | Lat | 2 | 346.6 | *<0.001** | 0.92 | 0.99 | P2>N1>P1 |
|  | Time | Amp | 2 | 0.7 | 0.493 | 0.06 | 0.17 |  |
|  |  | Lat | 2 | 1.9 | 0.158 | 0.02 | 0.38 |  |
|  | Group | Amp | 1 | 0.2 | 0.669 | 0.04 | 0.07 |  |
|  |  | Lat | 1 | 2.3 | 0.138 | 0.00 | 0.32 |  |
|  | Time x Group | Amp | 2 | 1.4 | 0.259 | 0.00 | 0.29 |  |
|  |  | Lat | 2 | 0.1 | 0.932 | 0.04 | 0.06 |  |
|  | Time x CAEP | Amp | 4 | 1.2 | 0.310 | 0.05 | 0.36 |  |
|  |  | Lat | 4 | 0.9 | 0.486 | 0.07 | 0.26 |  |
|  | Group x CAEP | Amp | 2 | 1.6 | 0.209 | 0.01 | 0.33 |  |
|  |  | Lat | 2 | 0.2 | 0.840 | 0.05 | 0.08 |  |
|  | Time x Group x CAEP | Amp | 4 | 0.2 | 0.918 | 0.05 | 0.10 |  |
|  |  | Lat | 4 | 0.8 | 0.553 | 0.02 | 0.23 |  |
|  | Error | Amp | 62 |  |  |  |  |  |
|  |  | Lat | 62 |  |  |  |  |  |
| **Cz** | CAEP | Amp | 2 | 2 | 0.000 | 0.563 | 1 | N1<P2<P1 |
|  |  | Lat | 2 | 2 | 0.000 | 0.884 | 1 | P2>N1>P1 |
|  | Time | Amp | 2 | 1.1 | 0.373 | 0.03 | 0.23 |  |
|  |  | Lat | 2 | 1.0 | 0.342 | 0.03 | 0.22 |  |
|  | Group | Amp | 1 | 0.2 | 0.660 | 0.00 | 0.07 |  |
|  |  | Lat | 1 | 1.0 | 0.311 | 0.02 | 0.17 |  |
|  | Time x Group | Amp | 4 | 0.7 | 0.570 | 0.05 | 0.22 |  |
|  |  | Lat | 4 | 0.7 | 0.624 | 0.04 | 0.20 |  |
|  | Time x CAEP | Amp | 2 | 0.6 | 0.548 | 0.02 | 0.15 |  |
|  |  | Lat | 2 | 1.8 | 0.178 | 0.05 | 0.36 |  |
|  | Group x CAEP | Amp | 2 | 1.7 | 0.185 | 0.05 | 0.35 |  |
|  |  | Lat | 2 | 2.6 | 0.083 | 0.08 | 0.50 |  |
|  | Time x Group x CAEP | Amp | 4 | 0.5 | 0.771 | 0.03 | 0.15 |  |
|  |  | Lat | 4 | 0.3 | 0.884 | 0.02 | 0.11 |  |
|  | Error | Amp | 62 |  |  |  |  |  |
|  |  | Lat | 62 |  |  |  |  |  |

| **Temporal site (T7, T8)** | CAEP | Amp | 2 | 23.3 | *<0.001** | 0.43 | 0.99 | Ta>Na, Tb |
| --- | --- | --- | --- | --- | --- | --- | --- | --- |
|  |  | Lat | 2 | 87.9 | *<0.001** | 0.74 | 0.99 | Tb>Ta>Na |
|  | Time | Amp | 2 | 0.6 | 0.572 | 0.07 | 0.14 |  |
|  |  | Lat | 2 | 0.4 | 0.652 | 0.06 | 0.12 |  |
|  | Group | Amp | 1 | 1.9 | 0.168 | 0.06 | 0.28 |  |
|  |  | Lat | 1 | 0.2 | 0.689 | 0.01 | 0.07 |  |
|  | Time x Group | Amp | 2 | 0.5 | 0.591 | 0.03 | 0.13 |  |
|  |  | Lat | 2 | 3.8 | *0.028** | 0.02 | 0.67 | Na at 12m: L > R |
|  | Time x CAEP | Amp | 4 | 2.0 | 0.101 | 0.11 | 0.58 |  |
|  |  | Lat | 4 | 0.3 | 0.880 | 0.05 | 0.11 |  |
|  | Group x CAEP | Amp | 2 | 1.4 | 0.259 | 0.03 | 0.29 |  |
|  |  | Lat | 2 | 1.4 | 0.264 | 0.04 | 0.28 |  |
|  | Time x Group x CAEP | Amp | 4 | 0.3 | 0.865 | 0.04 | 0.12 |  |
|  |  | Lat | 4 | 0.6 | 0.651 | 0.02 | 0.19 |  |
|  | Error | Amp | 62 |  |  |  |  |  |
|  |  | Lat | 62 |  |  |  |  |  |
| **Temporal site (P7, P8)** | CAEP | Amp | 2 | 17.8 | *<0.001** | 0.37 | 0.99 | Ta>Na, Tb |
|  |  | Lat | 2 | 129.9 | *<0.001** | 0.81 | 0.99 | Tb>Ta>Na |
|  | Time | Amp | 2 | 2.3 | 0.114 | 0.02 | 0.44 |  |
|  |  | Lat | 2 | 2.1 | 0.130 | 0.01 | 0.42 |  |
|  | Group | Amp | 1 | 4.1 | *0.047** | 0.03 | 0.52 | Na: L > R |
|  |  | Lat | 1 | 0.8 | 0.369 | 0.00 | 0.15 |  |
|  | Time x Group | Amp | 2 | 0.8 | 0.448 | 0.02 | 0.18 |  |
|  |  | Lat | 2 | 0.6 | 0.562 | 0.11 | 0.14 |  |
|  | Time x CAEP | Amp | 4 | 1.9 | 0.118 | 0.12 | 0.55 |  |
|  |  | Lat | 4 | 0.8 | 0.531 | 0.02 | 0.24 |  |
|  | Group x CAEP | Amp | 2 | 1.0 | 0.364 | 0.04 | 0.22 |  |
|  |  | Lat | 2 | 1.4 | 0.260 | 0.04 | 0.29 |  |
|  | Time x Group x CAEP | Amp | 4 | 0.7 | 0.585 | 0.02 | 0.22 |  |
|  |  | Lat | 4 | 0.3 | 0.879 | 0.04 | 0.11 |  |
|  | Error | Amp | 62 |  |  |  |  |  |
|  |  | Lat | 62 |  |  |  |  |  |
| **Mastoid site (M1, M2)** | CAEP | Amp | 2 | 50.3 | *<0.001** | 0.62 | 0.99 | N1(RP)>P1(RP), P2(RP) |
|  |  | Lat | 2 | 97.0 | *<0.001** | 0.76 | 0.99 | P2(RP)>N1(RP)>P1(RP) |
|  | Time | Amp | 2 | 7.9 | *0.001** | 0.20 | 0.94 | 6m, 12m > Base |
|  |  | Lat | 2 | 1.3 | 0.270 | 0.04 | 0.28 |  |
|  | Group | Amp | 1 | 11.2 | *0.001** | 0.15 | 0.91 | P1(RP) Base, 6m: L > R N1(RP) Base, 12m: L > R P2(RP) Base: L > R |
|  |  | Lat | 1 | 14.6 | *<0.001** | 0.19 | 0.96 | N1(RP), P2(RP): L > R |
|  | Time x Group | Amp | 2 | 2.5 | 0.094 | 0.07 | 0.48 |  |
|  |  | Lat | 2 | 0.6 | 0.529 | 0.02 | 0.15 |  |
|  | Time x CAEP | Amp | 4 | 3.6 | *0.010** | 0.19 | 0.85 | N1(RP): 6m, 12m > Base |
|  |  | Lat | 4 | 0.9 | 0.455 | 0.06 | 0.28 |  |
|  | Group x CAEP | Amp | 2 | 1.7 | 0.193 | 0.05 | 0.34 |  |
|  |  | Lat | 2 | 11.5 | *<0.001** | 0.27 | 0.99 | N1(RP): L > R |
|  | Time x Group x CAEP | Amp | 4 | 1.7 | 0.160 | 0.10 | 0.49 |  |
|  |  | Lat | 4 | 0.6 | 0.650 | 0.04 | 0.19 |  |
|  | Error | Amp | 62 |  |  |  |  |  |
|  |  | Lat | 62 |  |  |  |  |  |
