# Supplementary material for: Cortical reorganization after cochlear implantation for adults with single-sided deafness
Source: PLoS One. 2018 Sep 24;13(9):e0204402. doi: 10.1371/journal.pone.0204402 (PMC6152998; doi:10.1371/journal.pone.0204402)
Supplement: S2 Table — Italics indicate significant effects after Bonferroni adjustment for multiple comparisons (p<0.017). (DOCX) [file pone.0204402.s005.docx]

**S2 Table: Results of Mann-Whitney tests comparing GFP and CAEP amplitude and latency at each test sessions between NH and SSD-CI subjects.** Italics indicate significant effects after Bonferroni adjustment for multiple comparisons (p<0.017).

| NH vs. SSD-CI-L | | Amplitude | | | | | | Latency | | | | | |
| --- | --- | --- | --- | --- | --- | --- | --- | --- | --- | --- | --- | --- | --- |
|  |  | **0m** | | **6m** | | **12m** | | **0m** | | **6m** | | **12m** | |
|  |  | **U** | **p** | **U** | **p** | **U** | **p** | **U** | **p** | **U** | **p** | **U** | **p** |
| GFP | **P1** | 14.0 | 0.42 | 12.5 | 0.305 | 10.0 | 0.171 | 18.0 | 0.826 | 10.5 | 0.186 | 16.0 | 0.608 |
|  | **N1** | 19.0 | 0.943 | 17.5 | 0.769 | 10.0 | 0.171 | 13.0 | 0.336 | 10.0 | 0.164 | 14.0 | 0.417 |
|  | **P2** | 19.0 | 0.943 | 15.0 | 0.524 | 9.0 | 0.127 | 19.5 | 0.999 | 10.0 | 0.163 | 3.0 | *0.015** |
| Cz | **P1** | 16.0 | 0.622 | 16.0 | 0.622 | 5.0 | 0.295 | 17.5 | 0.769 | 17.5 | 0.825 | 18.5 | 0.883 |
|  | **N1** | 18.0 | 0.826 | 17.0 | 0.724 | 15.0 | 0.524 | 12.5 | 0.300 | 12.5 | 0.122 | 18.8 | 0.882 |
|  | **P2** | 10.0 | 0.171 | 9.0 | 0.127 | 7.0 | 0.065 | 18.0 | 0.826 | 18.0 | 0.883 | 11.5 | 0.240 |
| T8 | **Na** | 13.0 | 0.354 | 17.0 | 0.724 | 16.0 | 0.622 | 17.0 | 0.713 | 17.0 | 0.942 | 6.0 | 0.048 |
|  | **Ta** | 7.0 | 0.065 | 12.0 | 0.284 | 17.5 | 0.769 | 11.5 | 0.240 | 11.5 | 0.557 | 8.5 | 0.102 |
|  | **Tb** | 19.0 | 0.943 | 13.0 | 0.354 | 18.0 | 0.833 | 19.0 | 0.943 | 19.0 | 0.107 | 9.5 | 0.141 |
| P8 | **Na** | 12.0 | 0.284 | 11.0 | 0.222 | 20.0 | 0.999 | 12.5 | 0.304 | 12.5 | 0.607 | 18.5 | 0.884 |
|  | **Ta** | 16.0 | 0.606 | 15.0 | 0.524 | 17.0 | 0.724 | 10.0 | 0.163 | 10.0 | 0.354 | 14.5 | 0.464 |
|  | **Tb** | 14.0 | 0.435 | 20.0 | 0.999 | 18.0 | 0.833 | 6.0 | 0.048 | 6.0 | *0.010** | 14.0 | 0.420 |
| M2 | **P1(RP)** | 18.0 | 0.826 | 16.0 | 0.608 | 19.0 | 0.942 | 7.5 | 0.079 | 7.5 | 0.127 | 19.5 | 0.999 |
|  | **N1(RP)** | 12.0 | 0.272 | 17.0 | 0.724 | 6.0 | 0.045 | 8.0 | 0.091 | 8.0 | 0.067 | 0.0 | *0.002** |
|  | **P2(RP)** | 14.5 | 0.464 | 16.0 | 0.622 | 16.0 | 0.622 | 5.0 | 0.034 | 5.0 | 0.028 | 6.0 | 0.045 |
| NH vs. SSD-CI-R | | | | | | | | | | | | | |
| GFP | **P1** | 14.0 | 0.808 | 11.0 | 0.461 | 9.0 | 0.283 | 15.5 | 0.999 | 10.0 | 0.349 | 3.0 | 0.033 |
|  | **N1** | 15.0 | 0.933 | 11.5 | 0.496 | 8.0 | 0.214 | 8.0 | 0.200 | 0.0 | *0.008** | 10.5 | 0.394 |
|  | **P2** | 14.0 | 0.808 | 12.0 | 0.570 | 6.0 | 0.109 | 5.0 | 0.072 | 5.0 | 0.074 | 3.0 | 0.033 |
| Cz | **P1** | 4.0 | 0.048 | 16.0 | 0.999 | 10.0 | 0.368 | 15.5 | 0.999 | 10.0 | 0.345 | 12.5 | 0.607 |
|  | **N1** | 14.0 | 0.808 | 4.0 | 0.049 | 10.0 | 0.368 | 13.0 | 0.668 | 0.0 | *0.008** | 11.0 | 0.442 |
|  | **P2** | 1.0 | *0.008** | 4.0 | 0.049 | 12.0 | 0.570 | 10.5 | 0.392 | 0.5 | 0.304 | 9.5 | 0.307 |
| T7 | **Na** | 14.0 | 0.808 | 15.0 | 0.933 | 15.0 | 0.933 | 9.0 | 0.266 | 9.5 | 0.307 | 11.0 | 0.444 |
|  | **Ta** | 3.5 | 0.041 | 10.0 | 0.368 | 13.0 | 0.683 | 13.0 | 0.670 | 16.0 | 0.999 | 9.5 | 0.307 |
|  | **Tb** | 14.5 | 0.865 | 14.0 | 0.808 | 15.0 | 0.933 | 14.5 | 0.865 | 15.5 | 0.999 | 10.0 | 0.368 |
| P7 | **Na** | 10.0 | 0.368 | 11.0 | 0.461 | 13.0 | 0.683 | 9.0 | 0.266 | 12.5 | 0.607 | 13.0 | 0.670 |
|  | **Ta** | 9.0 | 0.283 | 12.0 | 0.570 | 13.0 | 0.683 | 12.0 | 0.570 | 13.0 | 0.683 | 11.5 | 0.495 |
|  | **Tb** | 13.0 | 0.683 | 8.0 | 0.214 | 12.0 | 0.570 | 10.0 | 0.349 | 10.5 | 0.394 | 14.0 | 0.798 |
| M1 | **P1(RP)** | 13.0 | 0.671 | 12.0 | 0.552 | 16.0 | 0.932 | 4.0 | 0.049 | 11.0 | 0.435 | 8.0 | 0.199 |
|  | **N1(RP)** | 6.0 | 0.109 | 8.0 | 0.214 | 10.0 | 0.368 | 6.5 | 0.126 | 6.0 | 0.105 | 5.0 | 0.073 |
|  | **P2(RP)** | 16.0 | 0.999 | 9.0 | 0.283 | 15.0 | 0.933 | 2.0 | 0.016 | 11.0 | 0.461 | 10.5 | 0.395 |
